# A five-year prospective study of fatigue in primary Sjögren's syndrome

**DOI:** 10.1186/ar3487

**Published:** 2011-10-13

**Authors:** Karstein Haldorsen, Ingvar Bjelland, Anne Isine Bolstad, Roland Jonsson, Johan Gorgas Brun

**Affiliations:** 1 Broegelmann Research Laboratory, The Gade Institute, Laboratory Bldg., Haukeland University Hospital, N-5021 Bergen, Norway; 2 Department of Clinical Dentistry - Periodontics, University of Bergen, Aarstadveien 17, N-5009 Bergen, Norway; 3 Department of Child and Adolescent Mental Health Services, Haukeland University Hospital, N-5021 Bergen, Norway; 4 Department of Clinical Medicine, University of Bergen, N-5020 Bergen, Norway; 5 Department of Rheumatology, Haukeland University Hospital, N-5021 Bergen, Norway; 6 Section for Rheumatology, Institute of Medicine, University of Bergen, N-5020 Bergen, Norway

## Abstract

**Introduction:**

Fatigue is prevalent in primary Sjögren's syndrome (pSS), and contributes to the considerably reduced health related quality of life in this disease. The symptom is included in proposed disease activity and outcome measures for pSS. Several studies indicate that there is an inflammatory component of fatigue in pSS and other chronic inflammatory rheumatic diseases. The purpose of this study was to investigate fatigue change in pSS in a longitudinal study, and explore whether any clinical or laboratory variables at baseline, including serum cytokines, were associated with a change in fatigue scores over time.

**Methods:**

A clinical and laboratory investigation of 141 patients fulfilling the American-European consensus criteria of pSS was undertaken in the period May 2004 to April 2005. Median time since diagnosis was 5.5 years. Examinations included the fatigue questionnaires: fatigue severity scale (FSS), fatigue visual analogue scale (VAS), functional assessment of chronic illness therapy - fatigue (FACIT-F) and medical outcome study short form-36 (SF-36) vitality, which were repeated in a follow-up investigation in January and February 2010.

**Results:**

A total of 122 patients (87%) responded at both time-points. Thirty-five percent of patients experienced a clinically significant FSS increase. On the group level, fatigue measures did not change except that there was a slight deterioration in SF-36 vitality score. High serum anti-Sjögren's syndrome A antigen (anti-SSA) showed weak associations with high baseline fatigue, and patients with increasing fatigue had lower baseline unstimulated whole salivary volume. Weak associations between increasing fatigue and serum immunoglobulin G (IgG), and the pro-inflammatory cytokine interleukin-17 (IL-17), were observed. Baseline sicca symptoms correlated with higher fatigue both at baseline and with increasing fatigue over time. Linear regression analysis did not identify any predictive ability of clinical or laboratory measures on fatigue change over time.

**Conclusions:**

Fatigue remained mainly unchanged over time. Using multivariate models did not reveal any clinical or laboratory predictors of fatigue change over time.

## Introduction

Primary Sjögren's syndrome (pSS) is a systemic rheumatic autoimmune disease targeting, in particular, exocrine glands, with eye and mouth dryness as classic symptoms. Extraglandular manifestations include arthritis, skin vasculitis and lymphoma [1]. Fatigue is a common complaint in pSS and other rheumatic disorders, as well as in malignancies and several other chronic conditions. Fatigue may be defined as "an overwhelming sense of tiredness, lack of energy, and feeling of exhaustion" [2]. Approximately 70% of pSS patients suffer from substantial fatigue [3,4], compared with about 20% of the normal population [5].

In rheumatic diseases, it is debated whether fatigue is independently associated with disease activity. In pSS the proposed disease activity measures Sjögren's Syndrome Disease Activity Index (SSDAI) and Sjögren's Systemic Clinical Activity Index (SCAI) include fatigue as well as other subjective health complaints, and fatigue was included among a suggested core set of outcome measures in pSS [6,7]. Possible direct or indirect causes of fatigue in pSS that have been suggested include chronic pain, hypothyroidism, and hypothalamus-pituitary-adrenal axis disturbances [8,9]. Rituximab treatment was associated with fatigue improvement in two small double-blind, randomised studies, the most extensive of which included 20 pSS patients receiving rituximab and 10 receiving a placebo [10,11]. This treatment also caused improved exocrine function. Another paper reported a weak negative correlation between plasma noradrenaline and a subscale of the Multidimensional Fatigue Inventory in pSS, a finding which may reflect a link between fatigue and autonomic nervous disturbances in this disease [12]. Recently, cerebrospinal IL-1 receptor antagonist (IL-1Ra) levels showed an association with fatigue in a pSS study [13].

We have only identified one previous study investigating fatigue in pSS longitudinally. A Swedish follow-up study assessed 47 patients with fatigue using a visual analogue scale (VAS) and 29 patients with the vitality domain of the Medical Outcome Study Short Form-36 (SF-36) at baseline and after five years [14]. Fatigue VAS did not change significantly, while the mean SF-36 vitality sub-score improved by 6.0 (*P *= 0.026). The study did not report any change in baseline predictors of fatigue over time. Only a few, low-sample studies have assessed serum cytokines in relation to fatigue or health related quality of life (HR-QOL) in pSS [3,15,16]. Serum levels of interleukin (IL)-1β, IL-2, IL-6, IL-10 and tumour necrosis factor (TNF) were found not to be associated with multiple dimensions of fatigue in a cross-sectional study with 60 pSS patients and 139 population based controls [3].

The aims of this study were to investigate fatigue in pSS patients in a follow-up study with a larger sample size and to explore whether any clinical or laboratory variables, including several cytokines at baseline, were associated with change in fatigue scores over time.

## Materials and methods

### Patients and clinical examination

The Bergen pSS cohort at present comprises 141 patients fulfilling the American-European consensus criteria [17], recruited from the out-patient registry of the Department of Rheumatology, Haukeland University Hospital. All patients underwent a new clinical and laboratory investigation in the period May 2004 to April 2005. These investigations included Schirmer's I test as a measurement of the lacrimal function, and unstimulated whole salivary excretion (UWS), in addition to subjective assessments of dry eyes and dry mouth during the past week on 100 mm VAS scales.

### Laboratory analyses

Standard haematological and immunological tests were carried out, including antinuclear antibodies (ANA), anti-SSA and anti-Sjögren's syndrome B antigen (anti-SSB) and IgG. These laboratory tests were performed in the routine hospital laboratory. ANA, anti-SSA and anti-SSB were analysed by ELISA. Anti-SSA and -SSB statuses were classified dichotomously; other serum and blood laboratory values had continuous values. Lip biopsy focus score was recorded from the medical files. Serum cytokines were previously analysed at our laboratory [18]. The assay comprises analyses of 25 cytokines:

IL-1β, IL-1 receptor antagonist (IL-1Ra), IL-2, IL-2 receptor, IL-4, IL-5, IL-6, IL-7, IL-8,

IL-10, IL-13, IL-15, IL-17, TNF, IL-12p40, interferon (IFN)-γ, IFN-α, granulocyte macrophage colony-stimulating factor (GM-CSF), monokine induced by IFN-γ (MIG), monocyte chemotactic protein-1 (MCP-1), IFN-γ-induced protein 10 kDa (IP-10), macrophage inflammatory protein (MIP)-1α, MIP-1β, eotaxin and Regulated upon Activation, Normal T-cell Expressed, and Secreted (RANTES). Serum cytokine values below the lower limit of detection were replaced with the lower limit values.

### Evaluation of fatigue

Among fatigue instruments used in rheumatic disease studies, including pSS, is the Fatigue Severity Scale (FSS), Functional Assessment of Chronic Illness Therapy - Fatigue (FACIT-F) and different VAS variants. FSS assesses functional issues during the preceding two weeks [19]. FACIT-F is a general fatigue measure with emphasis on daily life function [20]. SF-36 assesses different health aspects during the preceding four weeks [21]. The vitality domain of SF-36 has been used as a proxy measure of fatigue in several conditions. FSS and fatigue VAS are positive scales in that higher values mean higher fatigue levels, while FACIT-F and vitality have the opposite direction. In the present study, Norwegian versions of FSS, fatigue VAS, FACIT-F and SF-36 vitality were used. The SF-36 mental health domain was also recorded and included in the analyses to account for possible depression bias. Regarding fatigue VAS, patients were asked: "How have you experienced fatigue (tretthet; that is, "tiredness") during last week?", and the anchors were 0 mm = tiredness is no problem, and 100 mm = tiredness is a big problem. The questionnaires were initially completed face-to-face in connection with the clinical investigation in 2004 to 2005. At follow-up, postal questionnaires were sent to the trial participants in January and February 2010. Three patients were deceased and one had emigrated to an unknown location, thus 137 patients were sent questionnaires at this time. Patients not responding also received a postal reminder and new questionnaires. The study was approved by the Regional Committee for Medical and Health Research Ethics, and informed consent was obtained from all participants.

### Statistics

The difference in fatigue measures between follow-up and baseline was computed. To assess whether or not fatigue changed over time, paired *t*-tests were applied. Fatigue was compared with the following clinical and laboratory variables: serum cytokine concentrations, CRP, serum IgG, ANA, anti-SSA, anti-SSB, blood sedimentation rate, and Schirmer's test, UWS, focus score, VAS assessments of eye and mouth dryness, and pain. Comparisons with baseline fatigue were performed using Spearman's rank coefficient (rho). Regarding change in fatigue over time, these variables were compared with continuous fatigue differences (using Spearman's rho), and dichotomised differences (increased fatigue or not, using the Mann-Whitney U test). Associations between dichotomous variables were calculated using Fisher's exact test or McNemar's test. Hierarchical multiple linear regression was used to assess the ability of clinical and laboratory control measures to predict fatigue change over time, over and above any effect of socio-demographic factors. Two-sided *P*-values were computed, and *P*-values below 0.05 were considered statistically significant. Analyses were performed using PASW Statistics 18.0 (SPSS Inc., Chicago, IL, USA) and Prism 5 (GraphPad Software Inc., San Diego, CA, USA). Power calculations were performed using PS 3.0 [22].

## Results

### Study sample and data quality

Median age at baseline was 57.0 years (range 24 to 74), median time since onset of pSS symptoms was 13.0 years, median time since diagnosis was 5.5 years, and 95% of the patients were females. Non-responders at follow-up did not significantly differ from responders in these respects. Four patients used methotrexate, 25 used antimalarials, 9 used prednisolone, and 5 took other immunomodulatory drugs. Baseline clinical and laboratory data were available for all patients except for UWS, serum thyroxine and CRP (*N = *140), and focus score (*N *= 119).

One missing FACIT-F item at baseline, and six missing FACIT-F and four missing FSS items at follow-up, were imputed by the method of using the personal mean item score [23]. Also, eight fatigue or SF-36 items at follow-up were originally filled in at two neighbouring boxes, these were replaced with the mean value of the two. After this, baseline fatigue data were complete for the four fatigue measures, except a missing FSS score for one of the 141 patients. Valid sample sizes for fatigue differences were 122 (FSS) or 121 (fatigue VAS, FACIT-F and vitality).

### Cross-sectional findings at baseline

**Figure 1:**
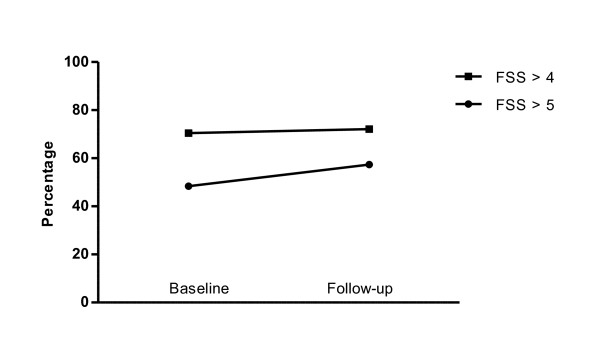
**Frequency of high fatigue**. Percentage of patients with high fatigue level, *N = *122. A high fatigue level defined as mean fatigue severity scale (FSS) score > 4 was experienced by 70.7% at baseline and 72.1% at follow-up (*P *= 0.86, McNemar's test). Using an FSS value of 5 as cut-off, the frequencies were 47.9% and 57.4%, respectively (*P *= 0.08, McNemar's test).

Table 1 shows baseline correlations between the four fatigue measures. Their absolute values varied between 0.47 and 0.75. This is a measure of convergent validity, which may be accepted as very good vis-à-vis FACIT-F (0.65 to 0.75), and good concerning the other measures. Defined by an FSS-score of more than 4, 70.7% of respondents reported high fatigue at baseline (Figure 1). Median (mean) FSS score was 5.00 (4.78) (interquartile range 3.67 to 6.22, SD 1.65).

**Table 1:** Pearson's correlation coefficients between fatigue measures at baseline

	FACIT-F	FSS	Vitality
Fatigue VAS	-0.65	0.47	-0.57
FACIT-F		-0.67	0.75
FSS			-0.51

*N = *140 for FSS comparisons, otherwise 141. *P *< 0.001 for all comparisons. Vitality and FACIT-F are negative scales, giving lower values with increasing fatigue. VAS, visual analogue scale; FACIT-F, Functional Assessment of Chronic Illness Therapy - Fatigue; FSS, Fatigue Severity Scale; vitality, short form-36 vitality domain.

Cross-sectional correlations with fatigue measures at baseline are shown in Table 2. A clear positive correlation between fatigue and pain was observed; moreover, there was a significant positive correlation with mouth dryness VAS. The SF-36 mental health domain (MH) was included in the analyses to account for possible depression bias. A low MH score was associated with higher fatigue and mean MH was slightly lower in patients with high fatigue defined by an FSS score > 4 (70.4 versus 76.7, *P *= 0.032). There were weak positive correlations between fatigue and age, and Schirmer's test. ANA showed a slight negative correlation with fatigue.

**Table 2:** Cross-sectional fatigue measures, significant correlations at baseline.

Correlating	Spearman's rho			
	
covariates	FSS	Fatigue VAS	FACIT-F	Vitality
Age			0.20*	
Mouth dryness VAS		0.31**	-0.20*	-0.20*
Pain VAS	0.32**	0.25**	-0.48**	-0.31**
Schirmer's test	0.18*			
ANA		-0.19*		
Mental health	-0.19*		0.32**	0.24**

N was 140 or 141 for the different comparisons. FACIT-F, vitality and mental health are negative scales (higher number means better health). *: *P *< 0.05; **: *P *< 0.01. Abbreviations: see Table 1.

A positive serum anti-SSA was over-represented among the high-fatigue patients (FSS > 4), with 68% of these versus 46% of the low-fatigue patients having anti-SSA (*P *= 0.023, odds ratio 2.42, 95% confidence interval 1.2 to 5.1). No cytokines in the 25-plex kit showed any association at baseline versus continuous fatigue measures and cytokine concentrations in serum did not differ in patients with high fatigue (FSS > 4) compared with patients having low fatigue. Dichotomising time since symptom start using the median of 13 years as a cut-off point did not reveal any difference in fatigue measures. Patients with one year or less since diagnosis (*N = *17) had slightly higher median FSS than the other participants (5.8 versus 5.0, *P *= 0.045). Regarding any influence of anemia or hypothyroidism causing fatigue, only one patient had a haemoglobin concentration below 9 g/l, and all had normal thyroxine levels. Five patients had elevated thyrotropin without concomitant lowered thyroxine, their FSS did not differ from the others (*P *= 0.93).

### Longitudinal univariate associations

At follow-up, 122 participants (87%) returned the fatigue questionnaires. Median follow-up time was 5.1 years (range 4.8 to 5.8). The proportion with high fatigue based on FSS did not change during the observation period (Figure 1). Using a minimal clinically important difference (MCID) for FSS of 0.6 [24], 35% of patients showed an FSS increase greater than this value. Floor-ceiling effects can potentially cause skewed results, if many patients have minimum or maximum scores so that calculated differences are curtailed. Twelve (9.9%) of the patients in the longitudinal analyses had the maximum FSS score of seven at baseline. Thirteen (10.7%) patients reached maximum FSS at follow-up, seven of these (5.7%) also had maximum FSS score at baseline. Furthermore, two and three patients had the lowest FSS score of one at baseline and follow-up, respectively. Regarding fatigue VAS and vitality, the frequency of minimum or maximum values varied from zero to four patients at either time point. FACIT-F data did not show floor-ceiling effects.

**Figure 2:**
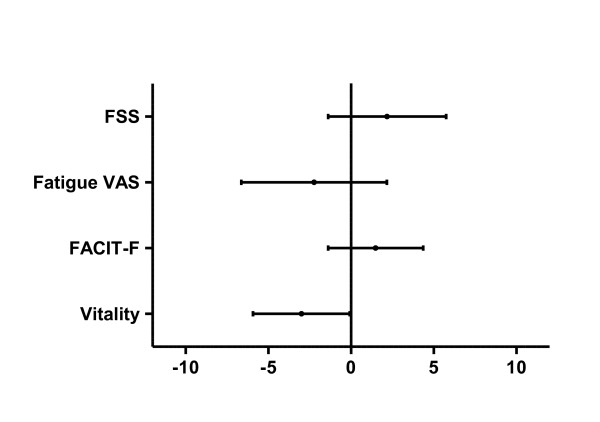
**Change in fatigue over time**. Errorbars showing 95% confidence intervals for the change in four different fatigue measures over time. *N = *122 for FSS, otherwise 121. The vertical line (zero) represents fatigue measures at baseline. FSS and FACIT-F data were normalised to a 0 to 100 scale, corresponding to fatigue VAS and vitality data. Mean change values (and standard deviations) without normalising were: FSS, 0.15 (0.12); fatigue VAS, -1.62 (2.20); FACIT-F, 0.78 (0.76); and vitality, -3.00 (1.48). Vitality (low values representing high fatigue) showed a statistically but not clinically significant decrease over time. The tendencies in the other measures were increasing fatigue by FSS and decreasing fatigue by VAS and FACIT-F. See Table 1 legend for definitions.

The difference in fatigue measures over time is illustrated in Figure 2, and the differences with standard deviations are given in the figure legend. Mean vitality showed a small but statistically significant worsening from 37.4 to 34.4 (*P *= 0.045, mean difference -3.00, 95% CI -5.92, -0.07). FSS, fatigue VAS and FACIT-F mean differences were contradictory regarding the direction of change, and their differences did not significantly deviate from zero.

Baseline clinical and laboratory data showed no significant correlation coefficients versus fatigue change over time, except FSS difference versus mouth dryness VAS (rho = 0.21, *P *= 0.02). Baseline MH showed no association with fatigue change over time. Dichotomising fatigue change into presence or absence of increased fatigue, showed several weak associations, though with relatively high *P*-values when the high number of comparisons is considered: baseline eye dryness VAS (*P *= 0.033) and mouth dryness VAS (*P *= 0.020) were associated with increasing FSS. Also, baseline UWS was somewhat higher in patients with FSS increase (*P *= 0.02), and IgG showed a slight association with decreasing vitality (*P *= 0.029). ANA showed a weak positive correlation with vitality decrease (*P *= 0.034), contradictory to the cross-sectional findings. Serum Il-17 (versus FSS increase, *P *= 0.020) and RANTES (versus vitality decrease, *P *= 0.047) were slightly higher among patients with increasing fatigue. CRP (*P *= 0.040) and IL-1β (*P *= 0.050) were lower in patients with increasing fatigue.

### Multivariate analysis

Hierarchical multiple linear regression was used to assess the ability of baseline clinical and laboratory control measures to predict fatigue change over time (Tables 3 and 4). Age, gender and education level were entered at Step 1 (education level had values from 1 = elementary school to 5 = university), Schirmer's test and UWS at Step 2, and focus score, anti-SSA/SSB, IgG and the SF-36 MH subscore at Step 3. The dependent variable was change in fatigue, and the model was repeated for all fatigue measures (change in FSS, FACIT-F, fatigue VAS and vitality). Step 1 (socio-demographic factors) predicted change in vitality, but no other partial model, and no final model, showed any significant prediction (Tables 3 and 4). The analysis was also performed with baseline mouth dryness, eye dryness and pain VAS added to Step 2, with unaltered results.

**Table 3:** Hierarchical multiple linear regression.

	FSS difference		FACIT-F difference		VAS difference		Vitality difference	
	Beta	*P*	Beta	*P*	Beta	*P*	Beta	*P*
Age	-0.10	0.42	-0.12	0.31	-0.11	0.37	-0.08	0.54
Education	0.04	0.69	-0.07	0.55	0.05	0.68	-0.19	0.08
Gender	0.03	0.79	-0.19	0.08	0.02	0.83	-0.08	0.50
Schirmer	-0.06	0.60	-0.10	0.37	0.07	0.55	-0.04	0.74
UWS	-0.05	0.65	-0.07	0.54	0.00	0.97	-0.08	0.47
Focus score	0.01	0.90	0.07	0.57	0.05	0.69	0.08	0.48
Anti-SSA	-0.11	0.44	-0.06	0.68	-0.02	0.90	0.00	0.99
Anti-SSB	-0.17	0.19	0.12	0.36	-0.13	0.34	0.10	0.46
IgG	0.09	0.46	0.00	0.98	-0.07	0.55	-0.09	0.45
Mental health	-0.03	0.81	0.03	0.78	0.02	0.86	-0.07	0.52
ANA	0.00	0.98	-0.01	0.93	0.06	0.70	-0.14	0.37

Standardised coefficients (beta) and *p*-values, one regression model for each fatigue measure. The difference of each fatigue measure is the dependent variable. *N = *103 for focus score data among the 122 follow-up patients, other variables had larger samples. The *P-*values for each model were 0.88, 0.66, 0.96 and 0.82, respectively. ANA, antinuclear antibodies; UWS, unstimulated whole saliva. FSS, VAS, FACIT-F, vitality, see Table 1 legend.

**Table 4:** Hierarchical multiple linear regression.

Independent variables	Change in fatigue measure			
	
	FSS	Fatigue VAS	FACIT-F	Vitality
Age, Gender, Highest education	0.014	0.021	0.051	0.094*
Schirmer, UWS	0.004	0.007	0.024	0.003
Focus score, anti-SSA, anti-SSB, IgG, MH	0.058	0.071	0.012	0.025

The table shows R square change values for each group of independent variables added in the regression model. R square change values signify the proportion of the total model variance that is explained by adding each of the variable groups. UWS, unstimulated whole saliva; MH, SF-36 mental health domain. Abbreviations, see Table 1 legend. * *P *= 0.047.

## Discussion

In 122 pSS patients, fatigue measured by FSS, FACIT-F, vitality and fatigue VAS showed no significant change during a mean follow-up time of 5.2 years, except a weak vitality worsening. The observed vitality worsening of 3.0 is not clinically significant according to a proposed MCID for vitality in systemic lupus erythematosus (SLE) of 10.7 [24]. Lack of association between time since symptom start and fatigue measures also supports a negative conclusion. Thirty-five percent of patients showed clinically significant deterioration, when measured by FSS increase greater than a proposed MCID of 0.6. FSS was higher, and not lower, in patients with the shortest time since diagnosis, which indicates that the fatigue due to pSS arises before diagnosis. In addition, there were no baseline clinical or laboratory predictors for fatigue change over time.

The increased fatigue in pSS and other rheumatic diseases may be comprised of an inflammatory and a psychosocial part [25]. Whether fatigue measures and assessment of other subjective complaints should be included in pSS disease activity and outcome measures is being discussed [7]. The main symptom change probably occurs early in the disease, and substantial diagnostic delay is rather the rule in pSS. In one study, mean time from first medical consultation to final diagnosis was nine years [26]; in our material, mean (median) time from symptom start to diagnosis was 6.0 (8.2) years.

It has also been difficult to show any reduction in exocrine function or HR-QOL over time [14,27]. Further, in sicca symptoms without inflammatory disease features, fatigue may be as prevalent as in pSS [28,29], which probably reflects a common psychosocial contribution to fatigue. Fatigue in rheumatic disease may largely be an unspecific sensation due to disease chronicity and damage. Fatigue measures do not distinguish between the effects of reversible inflammation and organ damage. In pSS, reduced exocrine function can strongly influence quality of life, and fatigue may be an unspecific part of this. Rituximab treatment was associated with fatigue improvement in two small double-blind, randomised studies [10,11]. This points to an attributable inflammatory component in fatigue in pSS, although such a conclusion could also be biased by improved exocrine function unspecifically causing less fatigue. In the present study, patients with one year or less since diagnosis had slightly higher median FSS than the other participants. This points to a plausible connection between higher disease activity in "early" pSS and, thus, higher fatigue, a hypothesis which should be explored in further studies using validated pSS disease activity measures.

Other researchers have found an association between depression and fatigue in pSS [4,12,30]. Depression does, however, not seem to be the primary cause of fatigue in this disease [4]. The SF-36 MH subscale, which mainly comprises depressive symptoms, showed lower values (more symptoms) in patients with high fatigue (FSS > 4). However, a fatigue change bias by mental symptoms as reflected in MH was not evident in the multivariate analyses.

In the cross-sectional analyses, a positive anti-SSA was over-represented among the high-fatigue patients (FSS > 4), with an odds ratio of 2.42. We also report a number of other minor findings, some of which may be incidental due to the high number of statistical comparisons. Regarding IgG, anti-SSA, and the pro-inflammatory cytokine IL-17, further studies should explore whether the findings can be replicated in pSS patient serum. Also, cytokine concentrations could preferably be analysed in cerebrospinal fluid, though this does not necessarily address neurologic changes because other mechanisms, for example, autonomic dysfunction, may be responsible for neurologic dysfunction in fatigue.

All in all, this study found mainly unchanged fatigue over time in pSS, as in the previous smaller study [14]. In addition, there was no reliable association with baseline salivary or lacrimal function, autoantibodies or focus score. The present work employed a higher sample size giving better power. Serum cytokines in pSS have not been investigated earlier in relation to change in fatigue over time, and this study includes a larger number of cytokines to explore possible links to the biologic correlate of fatigue.

Our study has some limitations. The limited sample size may have influenced our findings. Given the observed dispersion and a power of 80%, our data would permit the detection of a true mean difference in FSS, fatigue VAS and FACIT-F of 0.36, 6.27, and 8.30, respectively. Another issue is floor-ceiling effects, and 7 of the 122 patients had the maximum FSS score both at baseline and follow-up, possibly masking any change over time. By hypothetically changing these seven baseline FSS values to the lowest value of 1, the FSS difference would change from 0.15 to 0.50 (*P *= 0.006), which is still below the proposed MCID of 0.6 for FSS [24]. Baseline fatigue data were collected by questionnaires completed face-to-face. At follow-up, postal questionnaires were used, and this may have influenced or skewed the follow-up fatigue data.

## Conclusions

In 122 pSS patients, fatigue measured by FSS, FACIT-F, SF-36 vitality and fatigue VAS showed no significant change during a mean follow-up time of 5.2 years, except a weak but not clinically significant worsening in vitality score (*P *= 0.045). A total of 35% of patients experienced a clinically significant FSS increase. Multiple regression models showed no predictive ability of important clinical and laboratory variables on change in fatigue. In univariate analyses, high serum anti-SSA showed weak associations with high baseline fatigue, and patients with increasing fatigue had lower baseline UWS. Baseline sicca symptoms correlated with higher fatigue both at baseline and with increasing fatigue over time. Weak associations between increasing fatigue and serum IgG, and the pro-inflammatory cytokine IL-17, were observed. Although not consistently associated both at baseline and follow-up, further studies should explore whether these associations with laboratory variables can be replicated in pSS patient serum, and cytokine concentrations could preferably be analysed in cerebrospinal fluid.

## Abbreviations

ANA: antinuclear antibodies; FACIT-F: Functional Assessment of Chronic Illness Therapy - Fatigue; FSS: fatigue severity scale; GM-CSF: granulocyte macrophage colony-stimulating factor; HR-QOL: health-related quality of life; IFN: interferon; IL: interleukin; MCID: minimal clinically important difference; MCP-1: monocyte chemotactic protein-1; MH: SF-36 mental health domain; MIG: monokine induced by IFN-γ; MIP: macrophage inflammatory protein; pSS: primary Sjögren's syndrome; SCAI: Sjögren's Systemic Clinical Activity Index; SF-36: Medical Outcome Study Short Form-36; SLE: systemic lupus erythematosus; SSDAI: Sjögren's Syndrome Disease Activity Index; TNF: tumour necrosis factor; UWS: unstimulated whole saliva; VAS: visual analogue scale.

## Competing interests

The authors declare that they have no competing interests.

## Authors' contributions

KH participated in data collection, performed the statistical analyses and drafted the manuscript. IB participated in statistical analyses and in drafting the manuscript. JGB, RJ and AIB conceived of the study, participated in the data collection and helped to draft the manuscript. All authors read and approved the final manuscript.
